# What contributes to individual differences in brain structure?

**DOI:** 10.3389/fnhum.2014.00262

**Published:** 2014-04-28

**Authors:** Jenny Gu, Ryota Kanai

**Affiliations:** ^1^School of Psychology, University of SussexBrighton, UK; ^2^Sackler Centre for Consciousness Science, University of SussexBrighton, UK

**Keywords:** individual differences, brain structure, structural MRI, plasticity, gene environment interactions

## Abstract

Individual differences in adult human brain structure have been found to reveal a great deal of information about variability in behaviors, cognitive abilities and mental and physical health. Driven by such evidence, what contributes to individual variation in brain structure has gained accelerated attention as a research question. Findings thus far appear to support the notion that an individual’s brain architecture is determined largely by genetic and environmental influences. This review aims to evaluate the empirical literature on whether and how genes and the environment contribute to individual differences in brain structure. It first considers how genetic and environmental effects may separately contribute to brain morphology, by examining evidence from twin, genome-wide association, cross-sectional and longitudinal studies. Next, evidence for the influence of the complex interplay between genetic and environmental factors, characterized as gene-environment interactions and correlations, is reviewed. In evaluating the extant literature, this review will conclude that both genetic and environmental factors play critical roles in contributing to individual variability in brain structure.

Modern cognitive neuroscience has demonstrated that individual differences in adult human brain structure is a rich source of information about variability in a huge range of behaviors (Kanai and Rees, 2011). For instance, findings have shown that anatomical differences underlie variability in empathy (Banissy et al., 2012; Lai et al., 2012), political orientation (Kanai et al., 2011), time perception (Hayashi et al., 2014), sensitivity to pain (Emerson et al., 2014), working memory, and attention (Machizawa et al., 2010; Soto et al., 2014), moral values (Lewis et al., 2012) and numerical processing (Cappelletti et al., 2014; Krause et al., 2014). Such studies were made possible through the development of non-invasive structural magnetic resonance imaging (sMRI) techniques, which allow *in vivo* examination of differences in brain morphology between people. The two most commonly used approaches are voxel-based morphometry (VBM) and diffusion tensor imaging (DTI). Whereas VBM involves examining differences in grey matter (GM) volume, density and concentration at particular locations in the brain using spatially normalized sMRI images (Ashburner and Friston, 2000), DTI is used to detect individual differences in the integrity of brain white matter (WM) fibers reflecting variation in behavior (Johansen-Berg, 2010), through examining fractional anisotropy (FA), or the extent to which water diffusion along axons is uniform (Mori and Zhang, 2006). In addition to measuring diffusion anisotropy, other common measures which offer greater insight into brain WM structure include investigating mean diffusivity of water and diffusion perpendicular (radial diffusion) and parallel (axial diffusion) to WM fibers (Alexander et al., 2007).

With the growing evidence base highlighting phenotypic implications of variability in brain structure and advances in computational techniques, it has never been more scientifically relevant and interesting to explore what contributes to individual differences in brain structure. Findings from studies which have empirically examined the antecedents of morphological differences using various research designs and sMRI techniques support the notion that an individual’s brain architecture emerges as a complex dialog between their genes and environment, fuelling the growing suspicion that the age-old nature versus nurture dichotomy is too simplistic. As brain structure underlies much variation in behavior, studying it as an intermediate phenotype and gene-environment effects on morphology as a pathway is important, as this simplifies the challenge of linking specific gene-environment effects to phenotypes and deepens our insight into the potential causes of variation underlying typical and atypical behavior.

This review aims to evaluate the literature on whether and how genes and the environment contribute to individual differences in adult brain structure. It will first consider evidence from twin, genome-wide association, cross-sectional and longitudinal studies, to explore how genetic and environmental effects may separately and differentially contribute to brain morphology. Evidence for structural changes resulting from the interplay between genetic and environmental factors, characterized as gene-environment interactions and correlations, will then be reviewed. Although there is an abundance of research examining the prenatal and developing brain (e.g., Gilmore et al., 2010), in particular the influence of sensitive periods, a limited time during development in which environmental effects on brain structure is maximized (e.g., Knudsen, 2004), these are considered beyond the scope of this review, which will focus on evaluating research related to adult brain structure.

## GENETIC CONTRIBUTIONS TO INDIVIDUAL DIFFERENCES IN BRAIN STRUCTURE

The bulk of the evidence for the involvement of genes in determining variation in brain structure emerges from twin studies. The design is used to estimate heritability, or variance in particular brain regions that can be accounted for by genes (Wray and Visscher, 2008), by comparing sMRI images from monozygotic (MZ) and dizygotic (DZ) twins. As MZ and DZ twin pairs share 100 and 50% of their genes, respectively, and typically share environments, differences between regions in MZ twins is assumed to reveal environmental or gene-environment interplay effects and differences between MZ and DZ twin pairs can be interpreted as genetic differences.

Twin studies have demonstrated that variation in total brain GM and WM volumes is highly heritable (82–90% heritability within MZ pairs; Baaré et al., 2001). There is also increasing evidence that genetic contributions differ across focal brain areas. High heritability estimates have been found for thalamus (80%), caudate nucleus (88%) and putamen (69%) volumes (den Braber et al., 2013), WM density of the corpus callosum (82%), GM density of the occipital cortex (83%) and amygdala (55–80%; Hulshoff Pol et al., 2006), WM integrity of bilateral parietal (84–85%), frontal (55–74%) and left occipital (76%) lobes (Chiang et al., 2009), and GM density of frontal and linguistic cortices (95–100%; Thompson et al., 2001). Moderate-high estimates have been found for the hippocampus (40–70%; Sullivan et al., 2001; den Braber et al., 2013), moderate estimates for the nucleus accumbens volume (44–61%; den Braber et al., 2013) and low estimates for gyral patterning of the cortex (7–17%; Bartley et al., 1997) and GM and WM surrounding the lateral ventricles (up to 50%; Hulshoff Pol et al., 2006). Therefore, genetic factors appear to primarily contribute to individual differences in the morphology of evolutionarily recent structures involved in higher-order functions, such as attention, cognition, language, and visual processing. Further support for this assertion comes from studies linking heritability of frontal GM density with heritability of cognitive function (Thompson et al., 2001) and demonstrating evidence for genetic mechanisms underlying intellectual performance and WM integrity (Chiang et al., 2009).

Although such research furthers our understanding of the extent to which different structures in the brain are genetically determined, many twin studies have limitations which make it difficult to ascertain the accuracy of heritability estimates and preclude strong conclusions regarding genetic influences on brain structure. One such limitation is that sample sizes are typically quite small (e.g., 19 and 20 twin pairs in Bartley et al. (1997) and Thompson et al. (2001), respectively). Thus many twin studies lack the statistical power needed to accurately test for environmental effects. Additionally, twin studies measure overall genetic and environmental contributions rather than the influence of particular genes on individual differences in brain structure. A further critique concerns the twin study design, which makes many assumptions, for instance, that MZ and DZ twin pairs share similar environments (Charney, 2008). In reality, it is conceivable that as MZ twins tend to be treated more similarly (Loehlin and Nichols, 1976), this may cause them to have more similar characteristics and brain structure.

An approach which overcomes many of these flaws is the *Enhancing Neuroimaging Genetics through Meta-Analysis* (ENIGMA) project, which combines sMRI images from over 21,000 people with their genetic data gathered from genome-wide association studies (GWAS), containing information on over 500,000 common genetic variants. This project not only achieves the large sample necessary to detect moderate effect sizes and draw robust conclusions, but is able to highlight specific genetic variants responsible for differences in brain regions. The largest brain imaging study performed using data from the ENIGMA project identified the *rs7294919* genetic variant, which is associated with a decreased bilateral hippocampal volume of 1.2% per risk allele and the *C allele* of the *rs10784502* variant in the *HMGA2 gene*, which is linked to 0.5% larger intracranial volume and 1.3 higher IQ points per allele (Stein et al., 2012).

Altogether, twin studies and GWAS have demonstrated support for specific genetic variants contributing to individual differences in distinct brain areas. However, such research is not typically concerned with specific environmental effects on differences in brain structure. In order to study this, these findings need to be complemented with data from cross-sectional and longitudinal research, which examine structural plasticity, or the evolved ability of the adult brain to continuously alter its structure in response to environmental influences (Zilles, 1992).

## ENVIRONMENTAL CONTRIBUTIONS TO INDIVIDUAL DIFFERENCES IN BRAIN STRUCTURE

Cross-sectional studies have demonstrated through comparing cohorts of participants at a single time point that environmental influences, such as training or learning, are associated with variation in brain regions related to the type of learning. For example, compared to inexperienced control participants, experienced meditators have greater GM concentration in regions relevant to meditation, such as the right anterior insula, right hippocampus, and left inferior temporal gyrus (Hölzel et al., 2008), experienced musicians have greater GM volumes in motor, parietal and temporal regions (Gaser and Schlaug, 2003), London taxi drivers have greater posterior hippocampal GM volumes, which correlated with the number of years spent in the profession (Maguire et al., 2000), professional ballet dancers have decreased GM volumes in the left premotor cortex, putamen, and superior frontal gyrus and decreased WM volume in the corpus callosum (Hänggi et al., 2010) and academic mathematicians have greater GM density in the inferior parietal and left inferior frontal lobules (Aydin et al., 2007). Furthermore, even in non-expert adults samples, greater FA in the fornix correlated with better recollection memory (Rudebeck et al., 2009) and decreased FA along the pathway between the amygdala and ventromedial prefrontal cortex has been associated with higher trait anxiety (Kim and Whalen, 2009).

Although cross-sectional studies illustrate links between environmental influences and individual differences in brain structure, they provide insufficient evidence for the causal impact of environmental effects, as it is not possible to determine whether morphological differences are the cause or consequence of variation in experience. Longitudinal and intervention studies, which allow experience to be manipulated and subtle changes to be measured within individuals over time, provide stronger evidence for environment-induced changes in relevant brain regions (May, 2011).

Longitudinal studies using VBM and DTI [for a review, see May (2011)] have demonstrated greater GM concentration in the left hippocampus, posterior cingulate cortex, temporo-parietal junction, and the cerebellum in participants who completed an eight-week mindfulness meditation intervention but not in control participants (Hölzel et al., 2011), greater posterior hippocampal GM volumes in individuals who completed 4 years of training to become London taxi drivers relative to controls (Woollett and Maguire, 2011), GM increases in sensorimotor regions and the parieto-occipital junction following 40 h of golf practice (Bezzola et al., 2011), FA increases in individuals receiving eight weeks intensive memory training versus controls (Engvig et al., 2012) and GM increases in mid-temporal regions and the left posterior intraparietal sulcus in participants trained for 3 months in juggling versus controls (Draganski et al., 2004).

Altogether, evidence from cross-sectional and longitudinal studies supports environmental experience as a contributor to individual differences in brain structure. However, despite its advantage over cross-sectional designs, many longitudinal experiments contain flaws. One weakness is that studies typically lack an active control group, making it impossible to conclude that changes are not a general learning effect, but specific to the task (Thomas and Baker, 2013). Another limitation concerns the lack of group by time point statistical interactions being reported in many studies (Thomas and Baker, 2013); reporting only the presence of a significant effect of time in the experimental but not the control group is not decisive evidence for a specific learning effect (Nieuwenhuis et al., 2011). Future research should use rigorous experimental design and appropriate statistical tests, in order to advance our knowledge of environmental contributions to individual differences in brain structure.

Interestingly, although in need of improvement, many findings from cross-sectional and longitudinal studies mirror that of GWAS and twin studies in demonstrating support for some brain regions being more amenable to change than others. For example, the hippocampus and gyral patterning of the cortex, which are moderately heritable (Sullivan et al., 2001; Eckert et al., 2002), have been shown to undergo learning-induced changes (e.g., Hänggi et al., 2010; Hölzel et al., 2011). However, cross-sectional studies have also found phenotypic differences related to variation in frontal brain areas and the corpus callosum (e.g., Aydin et al., 2007; Hänggi et al., 2010), which are highly genetically determined (e.g., Hulshoff Pol et al., 2006). This apparent contradiction could be due to design limitations of existing studies, plastic changes in these regions being genetically determined (May, 2011), or experience-induced changes not being dependent on the degree of heritability of brain regions due to distinct mechanisms underlying environmentally and geneticallycontrolled structural modifications. When studying the contributions of nature and nurture in isolation, we can only speculate about their combined influence on focal brain regions. In order to construct a more lucid picture of this process, attention needs to be directed to research concerning the interplay between genes and the environment.

## GENE-ENVIRONMENT INTERPLAY AND INDIVIDUAL DIFFERENCES IN BRAIN STRUCTURE

**FIGURE 1:**
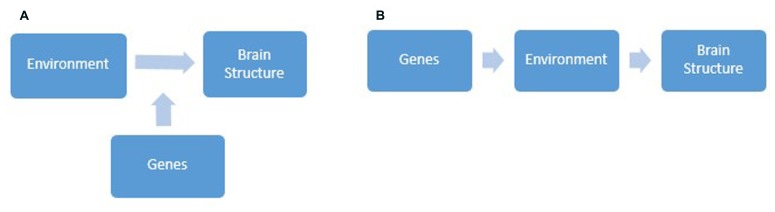
**Diagrams depicting gene-environment interaction **(A)**, whereby genes modulate individual differences in sensitivity to environmental influences and in turn brain structure, and gene-environment correlation **(B)**, whereby genes influence individual differences in the types of environment we are exposed to and in turn brain structure**.

Studies which combine measured genetic and environmental effects on brain structure are relatively new to cognitive neuroscience. Progress in this line of research is slowed by the presence of two interconnected but widely misunderstood concepts; *interaction* versus *correlation* between genetic and environmental influences (see **Figures 1A,B** for an illustration). The majority of studies have focused on studying gene-environment interaction (GxE), which is conceptualized as genetic control over individual differences in how *sensitive* we are to environmental influences (Plomin et al., 1977). Receiving less attention is gene-environment correlation (rGE), or genetic control of individual differences in *exposure* to environmental conditions (Plomin et al., 1977). rGE is split into three categories: passive, active, and evocative. Passive rGE arises from simply the genetic similarity between parents and their biological offspring. For example, children of musically gifted parents grow up being exposed to an environment with many musical references. Active correlation is associated with the tendency for individuals to seek environments consistent with their genetic predisposition. For instance, intelligent individuals might create situations that further enhance their intellectual ability. Finally, evocative rGE refers to differences in the reactions people elicit from their environments. For example, someone who displays manipulative behavior may be more likely to evoke high levels of negative affect from others. Although both types of gene-environment interplay are hugely interesting and worthy of study, reviewing research of all the possible ways in which genetic and environmental factors intertwine to influence brain morphology is beyond the scope of this review. This section will focus on evaluating GxE studies with reference to the wider context, in which GxE is but one process by which nature and nurture combine.

Geoffroy et al. (2013) recent systematic review identified two main ways in which GxEs influence variation in brain volumes in individuals diagnosed with schizophrenia (SZ). First, studies have found an interaction between genetic liability to SZ and pregnancy complications (PCs) on brain structure. For example, Haukvik et al. (2010) found that an allele variation in a SZ susceptibility gene, the *GRM3* gene (*rs13242038* variant), in combination with severe PCs (fetal hypoxia), increased hippocampal volume by on average 3.6% in both 54 SZ patients and 53 healthy control participants. Second, studies have demonstrated an interaction between genetic susceptibility to SZ and cannabis abuse, on brain WM volume. For instance, Ho et al. (2011) found using 235 SZ patients that heavy cannabis use coupled with the presence of genetic variants of cannabinoid receptor 1 contributed to decreased WM volumes in parietal lobes and impaired performance on a problem solving task. These findings give us a glimpse into how genetic predispositions, which modulate differences in *sensitivity* to environmental influences, may result in individual differences in brain structure and psychiatric phenotypes.

Despite these promising findings, the field of GxE is poorly explored, with most of the handful of extant studies focusing on established interactions in clinical populations. The additional presence of rGE slows progress in this area, as it suggests that factors we assume are “environmental” may in fact be under genetic control (Plomin et al., 1977). These correlations make it challenging to conceptually disentangle nature and nurture effects, to study the unique influences of genes, environment and GxE on brain structure. The existence of both GxE and rGE also questions the legitimacy of interpreting heritability estimates from twin studies, as it cannot be determined which genetic effects directly contribute to brain structure and which indirectly influence morphology, through controlling an individual’s sensitivity or exposure to environmental influences. This again underlines the importance of studying genetic and environmental influences together, in order to draw valid conclusions regarding how they contribute to individual differences in brain structure.

## CONCLUSIONS AND FUTURE DIRECTIONS

We reviewed the literature on genetic and the environmental contributions to individual differences in adult brain structure, by evaluating evidence from twin studies, GWAS, cross-sectional and longitudinal studies, and studies testing GxEs. Specifically, this paper demonstrated that both specific genetic factors and environmental effects are important contributors. Additionally, relatively novel GxE studies provide preliminary evidence for the role of genes in modulating differences in sensitivity to environmental influences, and in turn brain structure. As brain structure accounts for much variability in behavior, such research serves to further our understanding of the potential causes of typical and atypical behavior.

The priority for future research is to improve upon methodological limitations of existing studies in order to tease apart nature and nurture effects and present the strongest possible evidence for genetic and environmental contributions to individual differences in brain morphology. Other areas within this field which remain elusive include the specific microstructural processes involved in environment-induced structural plasticity. Findings from animal studies suggest changes may be mediated by neuronal remodeling (Lerch et al., 2011), synaptogenesis (Knott et al., 2006), or changes in axonal boutons (Yamahachi et al., 2009). Research into mechanistic pathways may also inform our understanding of the temporal parameters governing structural changes, such as, when they can first be detected and how long they last.

In conclusion, it is clear that both nature and nurture contribute to individual differences in brain structure. However, a consensus regarding the extent to which morphology is genetically or environmentally determined and the roles of GxE and rGE has not yet been reached. Although this line of research is fraught with complications, it is too early to declare these challenges insurmountable, as this is a relatively novel field and developments within it have been fast-paced. With the ever-increasing scale of genetic studies, increasing sophistication of computational techniques and plummeting costs of genotype sequencing (Wetterstrand, 2013), the prospects for successfully exploring how gene-environment associations contribute to individual differences in brain structure have never been brighter.

## Conflict of Interest Statement

The authors declare that the research was conducted in the absence of any commercial or financial relationships that could be construed as a potential conflict of interest.
